# Supplementary figures and images for: Determination of Triacylglycerol Composition in Mealworm Oil (Tenebrio molitor) via Electrospray Ionization Tandem Mass Spectrometry with Multiple Neutral Loss Scans
Source: Insects. 2024 May 17;15(5):365. doi: 10.3390/insects15050365 (PMC11121848; doi:10.3390/insects15050365)

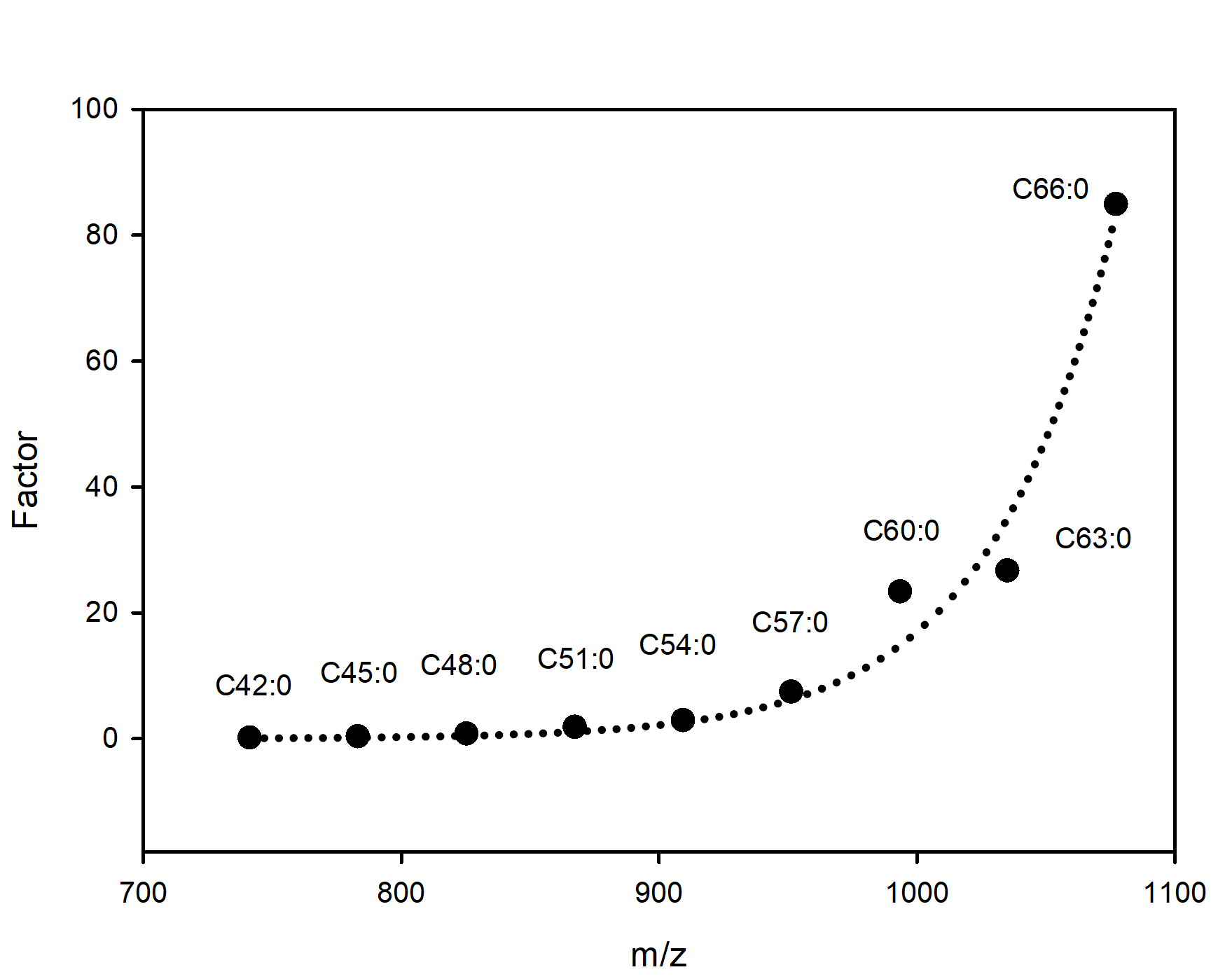

Supplement: Supplementary file 1 [file insects-15-00365-s001.zip › supplement_figure S1.JPG]

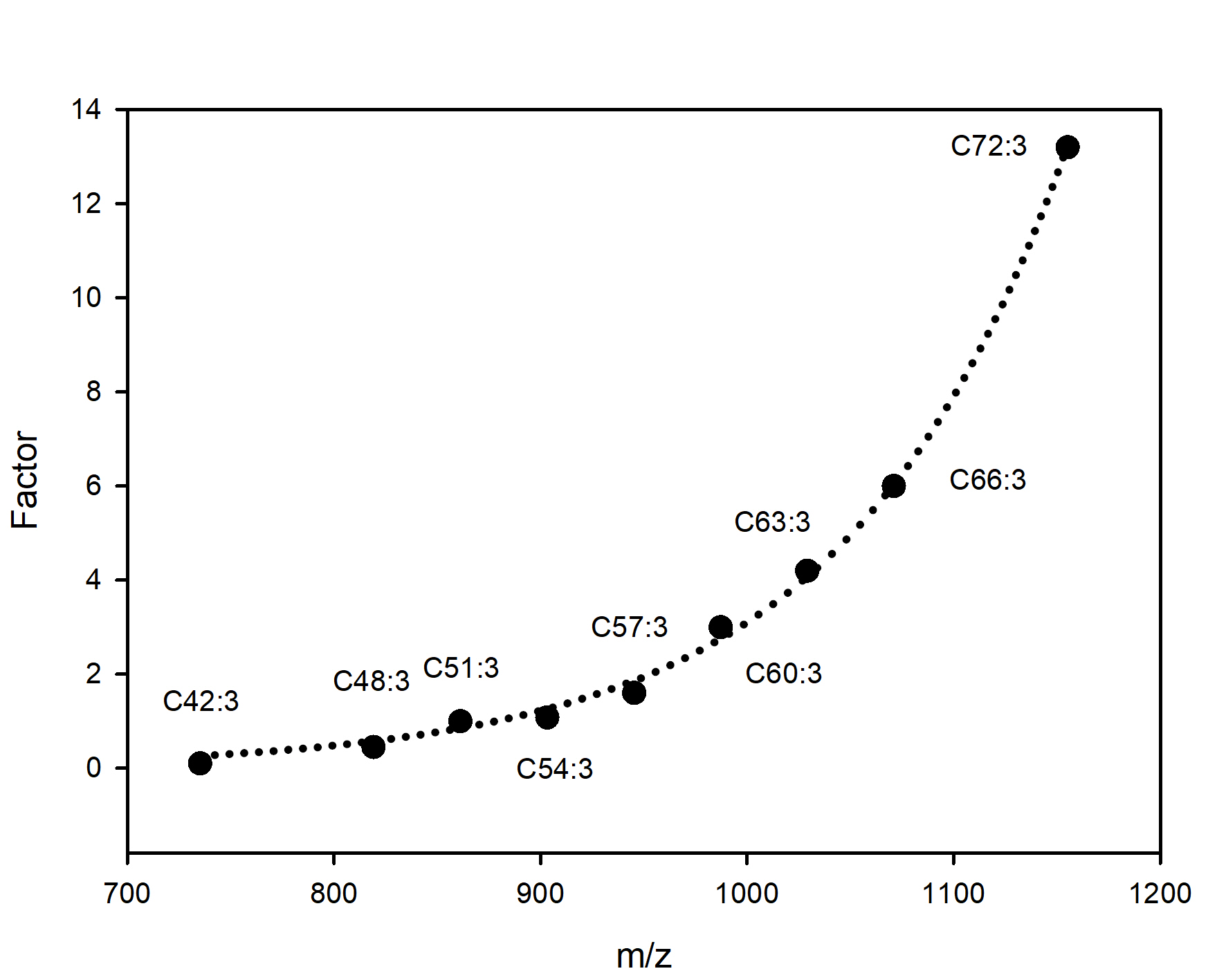

Supplement: Supplementary file 1 [file insects-15-00365-s001.zip › supplement_figure S2.JPG]

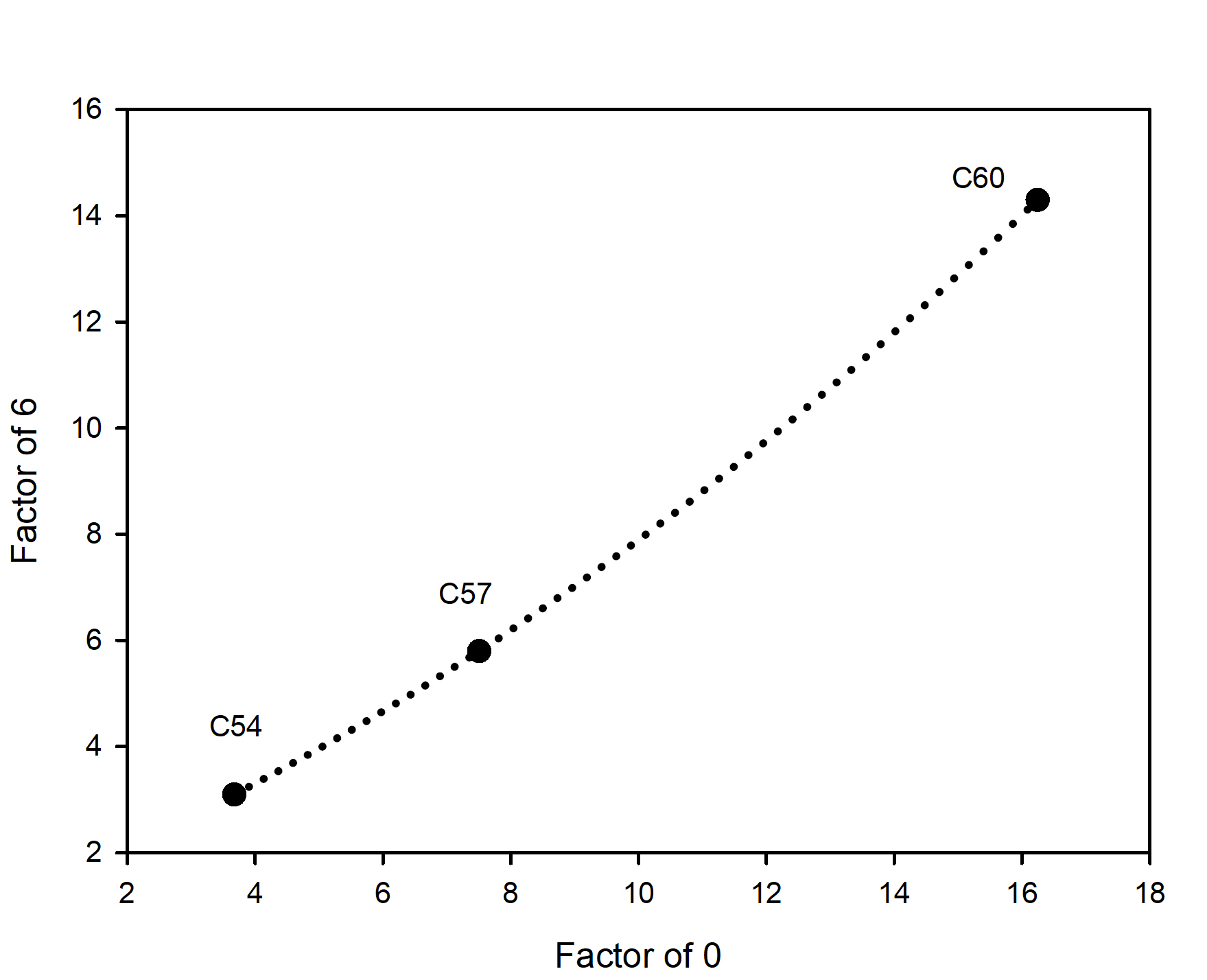

Supplement: Supplementary file 1 [file insects-15-00365-s001.zip › supplement_figure S3.JPG]

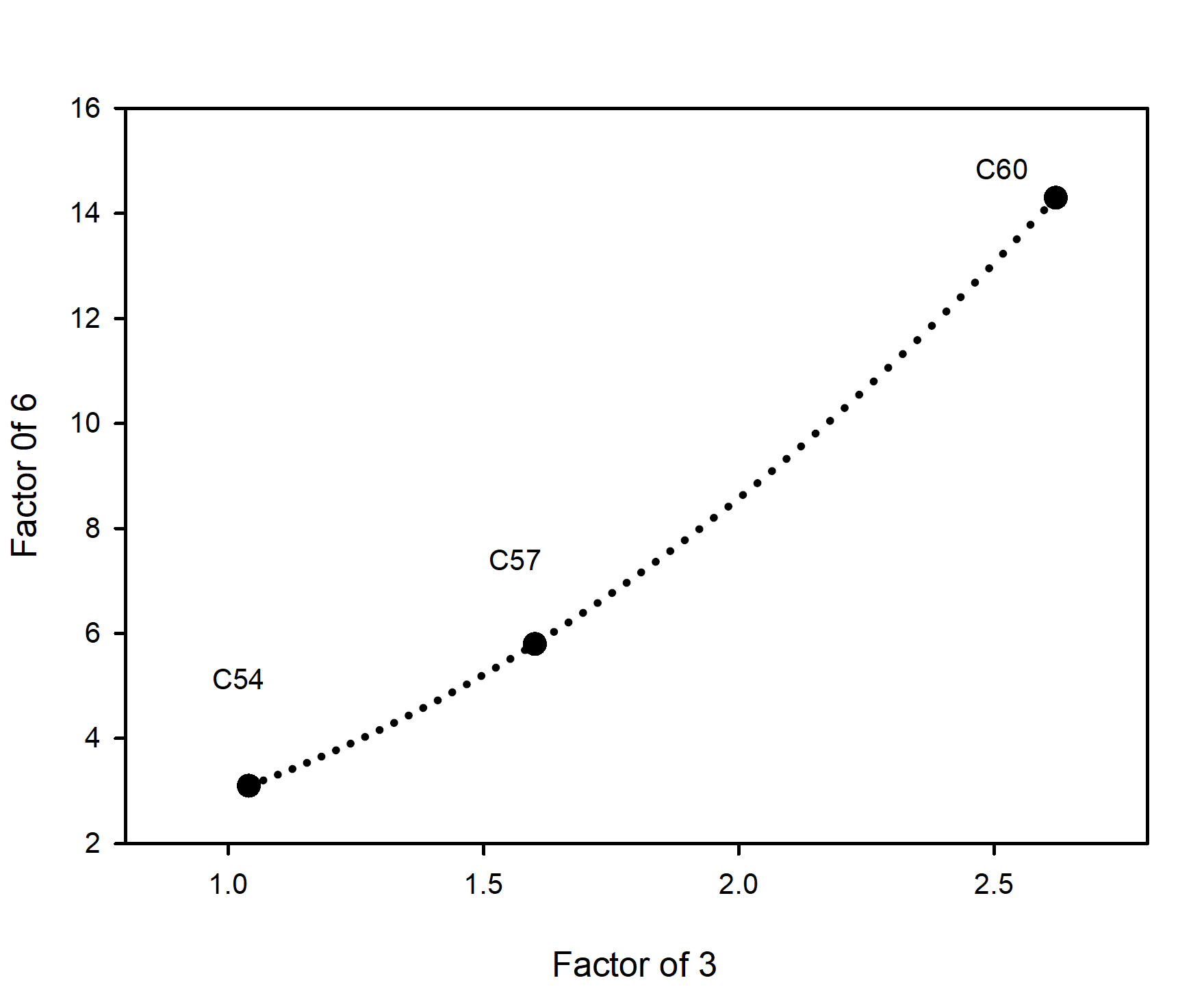

Supplement: Supplementary file 1 [file insects-15-00365-s001.zip › supplement_figure S4.JPG]
